# Numerical Investigation of an NACA 13112 Morphing Airfoil

**DOI:** 10.3390/biomimetics9100635

**Published:** 2024-10-18

**Authors:** Mădălin-Dorin Feraru, Daniel Măriuța, Marius Stoia-Djeska, Lucian-Teodor Grigorie

**Affiliations:** 1 Faculty of Aerospace Engineering, National University of Science and Technology Politehnica Bucharest, 060042 Bucharest, Romania; dorin.feraru@stud.aero.upb.ro (M.-D.F.); marius.stoia@upb.ro (M.S.-D.); 2 Department of Aircraft Integrated Systems and Mechanics, Military Technical Academy “Ferdinand I” Bucharest, 050141 Bucharest, Romania; daniel.mariuta@mta.ro

**Keywords:** IAR 330 Puma, trailing-edge flap, morphing actuation force

## Abstract

This article presents a numerical study on the 2D aerodynamic characteristics of an airfoil with a morphed camber. The operational regime of the main rotor blade of the IAR 330 PUMA helicopter was encompassed in CFD simulations, performed over an angle of attack range of α=[−3°; 18°], and a Mach number of M=0.38. Various degrees of camber adjustment were smoothly implemented to the trailing-edge section of the NACA13112 airfoil, with a corresponding chord length of c=600 mm at the Reynolds number, $Re=5.138\times 10^{6}$, and the resulting changes in static lift and drag were calculated. The study examines the critical parameters that affect the configuration of the morphing airfoil, particularly the length of the trailing edge morphing. This analysis demonstrates that increasing the morphed camber near the trailing edge enhances lift capability and indicates that the maximum lift of the airfoil depends on the morphed chord length. The suggested approach demonstrates potential and can be implemented across various categories of aerodynamic structures, such as propeller blade sections, tails, or wings.

## 1. Introduction

The contemporary sustainability objectives and green agenda concerning environmental challenges demand effective, innovative, and cost-efficient solutions within the aerospace sector [1]. Present investigative trends mainly focus on managing airflow and improving aerodynamic efficiency (either through drag reduction, lift enhancement, or both), which directly influence structural requirements, flight dynamics, energy consumption, and control, as well as flight safety and comfort. Progress in actuators, materials, sensors, and similar advanced technologies has led to breakthroughs that can considerably enhance aircraft safety, cost-effectiveness, and sustainability by exploring the potential of bio-inspired, smoothly transitioning aerodynamic designs. This has revived interest in morphing aircraft, moving away from traditional designs with hinges and pivots that have been in use for over a century [2,3].

The concept of adapting lifting structures, first observed in nature [4,5,6], presents a significant opportunity to enhance aircraft efficiency by dynamically adjusting configuration parameters, such as span, chord, and curvature, based on flight conditions. These morphing capabilities are directly inspired by biological systems, such as birds and fish, which modify their wing or fin geometry to optimize performance across varying environmental conditions. The application of morphing airfoils in engineering leverages these biological principles to improve both aerodynamic efficiency and operational functionality. Morphing airfoils are engineered to alter their geometry in real time, enabling superior adaptability across diverse flight regimes. By emulating the wing adjustments in birds or the fin reconfigurations in fish to manage lift and drag, these airfoils offer substantial aerodynamic benefits. Key structural modifications, such as variable camber and twist changes, replicate these biological adaptations, allowing the airfoil to respond to flow conditions and optimize lift-to-drag ratios dynamically. Advanced computational fluid dynamics (CFD) simulations are employed to model the interaction between structural deformations and aerodynamic forces, capturing the complex feedback mechanisms that are analogous to those found in biological systems. This biomimetic approach highlights how nature-inspired design principles can lead to breakthrough improvements in aerodynamic performance, rendering morphing airfoils both feasible and highly efficient for contemporary aviation applications [7,8].

For decades, initial passive methods, such as lift-enhancing devices (like slats and flaps) or winglets (designed to mitigate the adverse impacts of tip vortices), have been implemented on blades and wings, demonstrating their benefits [9,10]. These methods have also proven crucial in terms of control and noise generation [11]. Recent engineering and research efforts have increasingly concentrated on trailing edges and active leading capable of changing their configuration, as described in Akhter et al. [12]. Because of their heightened complexity, these solutions require structural, aerodynamic, and ultimately, aeroelastic evaluations [13]. As these variations mainly entail adjustments in the airfoil camber at the front and rear sections, their impacts are primarily observed in 2D analyses. This analysis is more fitting during the design phases, where optimization can also be integrated [14].

Renken conducted numerical simulations and wind tunnel tests to investigate a static-wing adaptable airfoil contour. The study revealed that optimizing the airfoil contour enhances aerodynamic effectiveness by aiming for smooth pressure distributions without shocks, which in turn reduces airfoil drag [15]. Szodruch and Hilbig showed that performance across various operational conditions for a fixed-wing aircraft with a variable camber wing can be improved by either decreasing fuel consumption by as much as 5.0% or increasing payload capacity [16]. Yeo utilized an extensive rotor analysis code to study the influences of various active manipulation techniques on rotor performance. In the investigated concepts, one aspect explored was the alteration of blade section camber through the implementation of static leading-edge droop and leading-edge slat mechanisms. Yeo’s findings indicated that in forward flight, the blade’s maximum loading capability was increased by these mechanisms for a morphing leading-edge camber. Furthermore, the overall rotor lift-to-drag ratio was improved by a dynamic 2/rev trailing-edge flap actuation. In other words, for every full revolution of the rotor, the trailing-edge flap is dynamically adjusted or actuated two times. This technique is used to enhance rotor performance by altering the flap’s configuration or position, optimizing the load distribution and reducing aerodynamic drag during flight [17]. Drag can be significantly reduced compared to a trailing-edge flap by implementing a consistent parabolic bending of the airfoil contour, as shown in the investigation of fixed-wing airfoils conducted recently by Hunsaker et al. [18]. Compared to a traditional flap, an airfoil with a constantly altering camber generated a negative shift in pitching moment for a specific variation in lift, as shown by the investigation. Additionally, this decrease was observed to be as much as 50% for high flap–chord ratios. The fish bone active camber (FishBAC) concept, proposed by Woods and Friswell, offers an alternative mechanism to conventional trailing-edge flaps for implementing camber alteration, enabling substantial and continuous morphing of the airfoil camber [19].

Trailing edge camber morphing is commonly favored for rotorcraft applications. Additionally, the overall morphing scheme incorporates the trailing edge camber morphing concept, initially conceived for fixed-wing aircraft. Because of its simplicity, utilization of conventional materials, and compatibility with off-the-shelf actuation options, this concept emerges as an excellent candidate for design and feasibility studies in rotorcraft. The force necessary to actuate a morphing trailing edge depends on several factors, including the aerodynamic forces acting on the surface, the rigidity of the materials used, and the choice of actuation system. Consequently, this study delves into the potential implementation of the idea of altering trailing-edge camber for rotorcraft [20,21,22].

**Figure 1:**
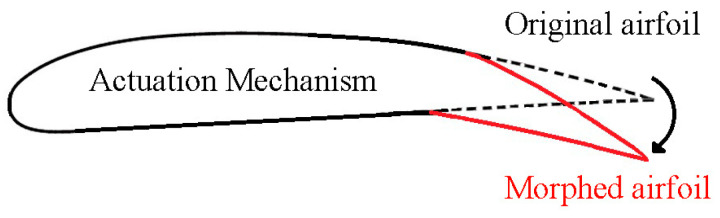
The proposed operational principle of TRIC for rotorcraft.

Figure 1 illustrates the operational principle of the translation-induced camber morphing (TRIC) concept designed for rotorcraft. It consists of a hollow trailing edge portion, while the remaining segment of the blade is constructed and derived from a traditional rotor blade design. An actuation system can transform this hollow trailing edge section. Where required, a blade can incorporate a trailing-edge flap and the appropriate actuation mechanism. Despite changes to the mass distribution and dynamic response of an existing blade, the TRIC morphing concept’s modular design provides benefits for inspection and maintenance. An efficient morphing mechanism can be developed by incorporating the necessary actuators and suitable trailing-edge adjustments [23,24,25,26,27].

Findings regarding a comprehensive analysis of the influence of TRIC on the aerodynamic characteristics of airfoils are provided and deliberated upon. The aerodynamic data for the 2D airfoil, represented in polar diagrams, that were produced will be utilized for thorough analyses of rotorcraft, incorporating dynamic camber morphing in a rotor system.

**Figure 2:**
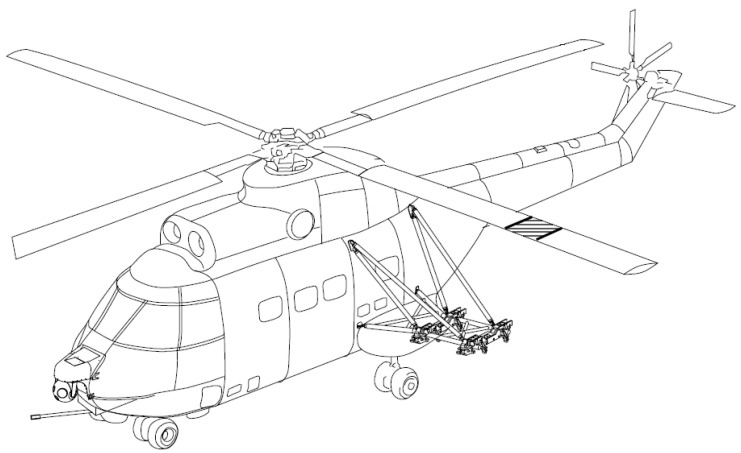
IAR 330 PUMA military helicopter [28].

This study examines the aerodynamic performance of the “Industria Aeronautica Romana” (IAR) 330 PUMA military helicopter rotor blade (see Figure 2) in hover flight at Mach number M=0.38. The rotor blade features different airfoil sections along its span (s): from s=0.25R to s=0.7R, it uses the NACA13112 airfoil; from s=0.7R to s=0.9R, it uses the NACA13109 airfoil; and from s=0.9R to the tip (R), it uses the NACA13106 airfoil.

To optimize the performance and efficiency of the rotor system, various combinations of actuation strategies, morphing mechanisms, and deflection targets need to be investigated. Before developing an efficient morphing mechanism, it is crucial to first calculate the actuation forces required to deform the morphing surface. This calculation provides key insights into the magnitude of the forces needed to adjust the trailing edge under various operating conditions. By understanding these actuation forces, the selection of the most suitable actuator—whether piezoelectric, hydraulic, or electromechanical—can be made. Once the actuation requirements are clear, an efficient morphing mechanism can be developed by incorporating the necessary actuators and suitable trailing-edge adjustments. For significant results at small deflection angles, the blade requires a larger internal space, and the section should be located closer to the tip. For this reason, the analyzed blade section features a NACA13112 airfoil, positioned at y=4225 mm, with a length of Δy=500 mm and a chord length of c=600 mm. Utilizing 2D CFD simulations and the NACA13112 airfoil as the standard, various airfoil configurations with varying degrees of camber morphing were investigated at M=0.38 and across a range of angle of attack (AoA) variations typical for this rotor [28].

## 2. Methods and Boundary Conditions

It is crucial to establish boundary conditions that can significantly impact the overall numerical study. For example, specific characteristics related to a camber morphing solution must include parameters, such as operational speed, frequency, and sizing. In the scenario being considered, the aerodynamic characteristics of the airfoil are outlined in Table 1.

### 2.1. Analytical Method

**Figure 3:**
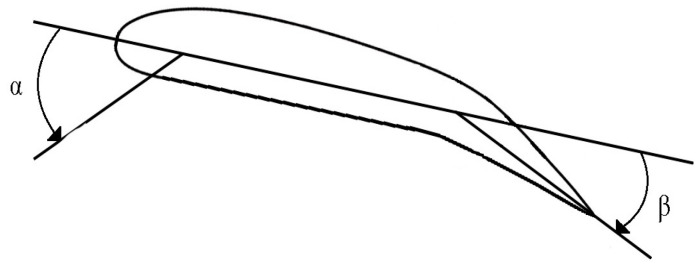
A control surface on a 2D airfoil.

In the depicted 2D configuration in Figure 3, the AoA α is the angle between the chord line (a straight line from the leading edge to the trailing edge of the airfoil) and the relative airflow. The angle of deflection β is the deflection angle at the trailing edge of the airfoil, which may be influenced by mechanisms, like trailing-edge flaps or camber changes. The AoA is crucial in determining the lift and drag generated by the airfoil. It is observable that increasing β leads to an augmentation in the lift coefficient $C_{l}$ while maintaining a constant slope for each curve. Thus, the implementation of the control surface enhances the effective camber of the airfoil [29].

**Figure 4:**
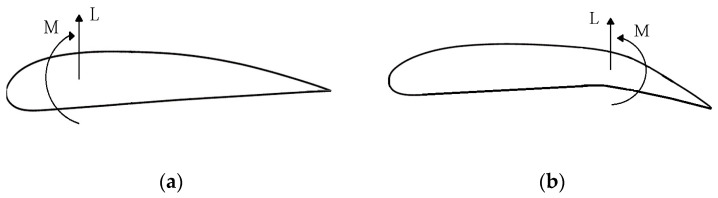
Effect on lift distribution of applying a control surface rotation: (**a**) the lift and aerodynamic moment generated by the helicopter rotor blade; (**b**) the lift and hinge moment provided by the flap.

Consequently, employing the control surface results in increased lift but introduces an extra nose-down pitching moment (see Figure 4).

**Figure 5:**
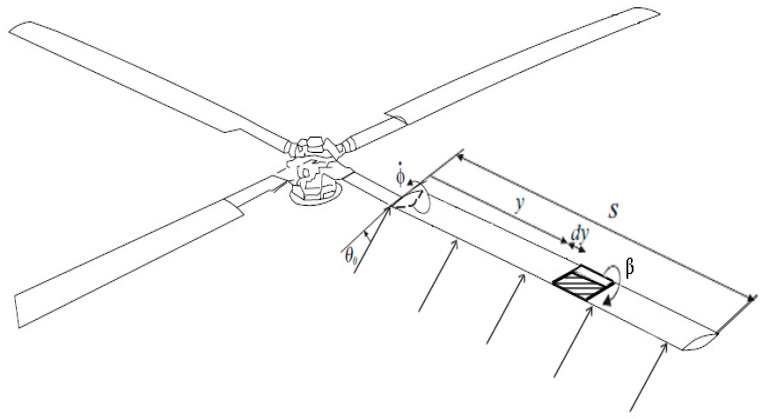
The rotor of the IAR 330 PUMA helicopter.

The rotor of the IAR 330 PUMA helicopter is examined (see Figure 5). It is characterized by a blade length (s) and chord length (c). The airfoil is symmetric, resulting in no lift generation and null incidence. The elastic axis is situated at a distance c·e from the axis of the aerodynamic center, where e represents the non-dimensional distance between the elastic axis and the aerodynamic center. The blade possesses torsional stiffness represented by GJ, where G is the shear modulus and J is the torsional moment of inertia. The incidence at the fixed end is indicated by $\theta_{0}$. The control surface is assumed to be rigid, with deflection angles measured positively downwards. Additionally, the rolling channel speed is assumed to be zero ($V_{R}=0$).

The twist is defined as nose-up about the flexural axis, taken at distance c·e aft of the aerodynamic center on the quarter chord. Let uss assume that the blade exhibits flexibility in twist, which is considered to vary linearly as follows:

(1) $$\theta =\left(\frac{y}{s}\right)\theta_{T}.$$

The lift and moment will depend on both the characteristics of the blade as well as those of the control surface. Therefore, $C_{l}$ can be expressed as follows:

(2) $$C_{l}=C_{l0}+C_{l\alpha }\alpha +C_{l\alpha_{c}}\beta ,$$

where

(3) $$C_{l0}=a_{0},$$

(4) $$C_{l\propto }=a_{w},$$

(5) $$C_{l\alpha_{c}}=\frac{\partial C_{lc}}{\partial \alpha }={\;a}_{c}.$$

This will result in the fact that:

(6) $$\;C_{l}=a_{0}+a_{w}\alpha +a_{c}\beta .$$

For the moment coefficient, it can be expressed in a similar manner, as follows:

(7) $$C_{m}=C_{m0}+C_{m\alpha }\alpha +C_{m\alpha_{c}}\beta ,$$

where

(8) $$C_{m0}=b_{0},$$

(9) $$C_{m\propto }=\frac{\partial C_{m}}{\partial \alpha }=b_{w},$$

(10) $$C_{m\alpha_{c}}=\frac{\partial C_{mc}}{\partial \alpha }={\;b}_{c}.$$

This will result in the fact that:

(11) $$\;C_{m}=b_{0}+b_{w}\alpha +b_{c}\beta .$$

Considering that the airfoils are symmetric, $a_{0}=b_{0}=0$. Furthermore, by definition, $b_{w}=a_{w}e$. For studying the effect of control surface deflection, the incidence at the fixed end is considered: $\theta_{0}=0$. Considering the form of $C_{l}$, the elemental lift and moment can be written as follows:

(12) $$dL=qcdy\left(a_{w}\frac{y}{s}\theta_{T}+a_{c}\beta \right),$$

(13) $$dM=qc^{2}dy\left(b_{w}\frac{y}{s}\theta_{T}+b_{c}\beta \right).$$

### 2.2. Panel Method

With the advent of digital computers, a compelling alternative to analytical methods has emerged, emphasizing numerical solutions. This approach hinges on the placement of singularities on individual sections of the airfoil surface. The unknown singularity strengths are calculated by establishing a set of linear algebraic equations that fulfill the Kutta condition and the no-penetration condition. Lift coefficients and pressure distribution can be readily predicted. Airfoil sections of any camber and thickness can utilize the panel method. In the preliminary stage, panel methods provide a reasonable level of accuracy and are less time-consuming. Thus, understanding their principles is crucial for future applications due to these benefits [30,31,32,33,34].

A key distinguishing feature of panel methods is the type of singularity element used to model the flow field around the airfoil, whether it is a source, doublet, or vortex element. Furthermore, the sensitivity of the results to the number of panels (N) is analyzed, showing that the accuracy of the results is particularly influenced by the choice of N.

A NACA 5-digit airfoil is characterized by several parameters, with the most significant ones being the maximum camber as a percentage of the chord (m), the location of the maximum camber along the chord (n), and the maximum thickness as a percentage of the chord (xx). This study utilizes the NACA13112 airfoil, where m=0.01 (1% maximum camber), p=0.31 (maximum camber located at 31% of the chord length), and xx=0.12 (12% maximum thickness).

Discretizing the airfoil contour into a number of panels is an integral part of the panel method. Control points are positioned at the midpoint of each panel, with each panel characterized by a linear source distribution. Specific formulas for the panel method are then used to compute the impact of each panel on these control points. The important equations used in the calculations are as follows:

1. Influence coefficient equation:
  (14) $$A_{ij}=\frac{\theta_{2j}-\theta_{1j}}{2\pi },$$
  for i≠j, where $\theta_{1j\;}$ and $\theta_{2j\;}$ are the angles subtended by the endpoints of the panel at the corresponding control point. This equation represents the influence of the j-th panel on the i-th control point;
2. Kutta condition:
  (15) $$\gamma_{1}+\gamma_{n}=0.$$

This condition ensures that the circulation at the trailing edge is zero, preventing velocity and pressure jumps;

3. Linear system of equations:
  (16) Aγ=b.

Matrix A is an n×n matrix where the diagonal elements (i=j) might require special treatment because they represent the influence of a panel on itself, and the off-diagonal elements (i≠j) are calculated using the influence coefficient equation. For an airfoil discretized into N panels, A is as follows:

(17) $$A=\left[\begin{array}{c} \;A_{11}\\ A_{21}\\ \vdots \\ A_{n1} \end{array}\begin{array}{c} \;A_{12}\\ A_{22}\\ \vdots \\ A_{n2} \end{array}\begin{array}{c} \cdots \;\\ \cdots \\ \ddots \\ \cdots \end{array}\begin{array}{c} A_{1n}\;\\ A_{2n}\\ \vdots \\ A_{nn} \end{array}\right],$$

where each element $A_{ij}$ is calculated using the influence coefficient equation. The vector γ represents the unknown circulation densities at each panel, and it has the following form:

(18) $$A=\left[\begin{array}{c} \gamma_{1}\\ \gamma_{2}\\ \vdots \\ \gamma_{n} \end{array}\right].$$

These values are what we solve for to determine the flow characteristics around the airfoil. The vector b contains the free-stream velocity contributions, which depend on the AoA α. For each control point, the term is typically related to the angle between the incoming flow and the panel, as well as any boundary conditions. It is depicted as follows:

(19) $$b=\left[\begin{array}{c} b_{1}\\ b_{2}\\ \vdots \\ b_{n} \end{array}\right],$$

where each $b_{i}$ is calculated based on the geometry of the panel and the AoA, as follows:

(20) $$b_{i}=-V_{\infty }\cos\left(\theta_{i}-\alpha \right),$$

where $V_{\infty }$ is the free-stream velocity, and $\theta_{i}$ is the angle of the panel relative to the free-stream direction;

4. Calculation of velocity and pressure coefficient:
  (21) $$V_{i}=\cos\left(\theta_{i}-\alpha \right)+\sum_{j=1}^{n}\frac{\gamma_{i}}{2\pi }\frac{\left(\theta_{2j}-\theta_{1j}\right)}{{∆}_{s_{j}}},$$
  (22) $$C_{p}=1-V^{2}.$$
  where ${∆}_{s_{j}}$ is the length of the j-th panel, $C_{p}$ is the pressure coefficient, and $V_{i}$ is the velocity at the control points.

### 2.3. CFD Method

The initial objective, essential for establishing any analysis, was to identify a technique to alter the NACA13112 airfoil. The literature showcases camber morphing airfoils through different methods [35,36,37,38,39]. One approach to obtaining morphed aerodynamic airfoils involves using a MATLAB code package from the University of Bristol [40]. This package is employed to modify the camber of the baseline airfoil across the rear quarter chord and to create new airfoil configurations for further examination. It utilizes a third-order polynomial morphing function to establish the new camber based on specified trailing-edge tip deflections, expressed as a proportion of the baseline chord. Due to practical constraints in affecting camber changes near the trailing edge, the morphing function can only be defined up to 95% of the chord length [41].

Manually altering the airfoil’s coordinates to replicate a modified trailing edge is a highly labor-intensive task. In this paper, the airfoil coordinates were imported into CATIA Generative Shape Design [42,43]. The airfoil section was extended to create a surface. This surface was then modified to replicate a control surface deflection through the use of the SHAPE MORPHING tool available in CATIA V5 R21. Once the surface had been altered, a 2D section of the morphed airfoil was projected. This morphed airfoil configuration was utilized to generate a 2D surface, which was subsequently imported into the ANSYS Fluent Workbench. ANSYS Fluent was chosen for its versatility in handling aerodynamic simulations, particularly in scenarios involving complex geometrical changes and airflow dynamics [43,44].

**Figure 6:**
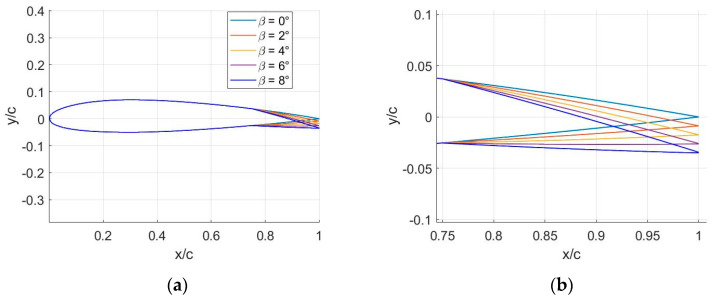
(**a**) Various camber morphing configurations for the NACA13112 airfoil with positive deflection of the morphed flap; (**b**) zoomed-in view of the morphing trailing edge.

The morphed configurations, while generically representative of the TRIC mechanism, maintained identical surface contours to the standard airfoil NACA13112 from the leading edge to the chord fraction cf=0.75c. All examined airfoil configurations ensure consistency with c=600 mm for the standard airfoil utilized for the primary rotor of the IAR 330 PUMA military helicopter. The degree of camber morphing varied between β=[2°, 4°, 6°, 8°], while β=[0°] represents the baseline airfoil configuration (see Figure 6).

Using CFD for incompressible flow analysis, a computational aerodynamics investigation was conducted to evaluate the 2D aerodynamic performance of the specified morphed airfoil configuration derived from the NACA13112 airfoil [45,46,47,48,49,50]. The flow was assumed to be completely turbulent during the entire analysis. CFD analyses were conducted at M=0.38 over a range of AoA from α=−3° to α=18°. Although the primary focus of this study is on positive deflections of the morphed flap, the CFD simulations were executed by varying β within the range of [−4°;8°], in intervals of β=2°, to mitigate computational complexity. Each morphed configuration was generated following the previously outlined procedure [51,52,53]. Abdelmoula et al. also noted similar observations when they studied the camber morphing airfoils at M=0.4 [54].

A structured C-grid mesh was utilized to construct the computational domain. A grid convergence study was performed using various grid resolutions for the baseline case and a camber morphing case β=8° at M=0.38, to establish the grid uncertainty. When adaptive sizing was applied, the number of cells increased significantly, especially in critical regions of the flow, such as around the leading and trailing edges of the airfoil, as well as near the airfoil surface where boundary layer effects are dominant. However, adaptive sizing substantially increased the computational time, and since it did not lead to improved accuracy, it was not used in the subsequent analyses presented in this study. Table 2 outlines the different grid resolutions employed for the grid convergence study.

**Figure 7:**
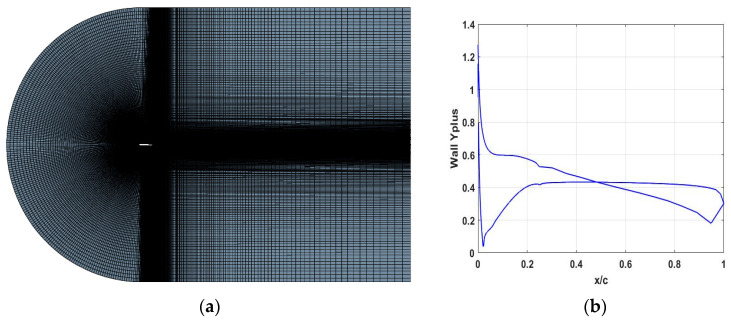
Mesh used in this particular investigation: (**a**) C-grid mesh; (**b**) the dimensionless wall distance $y^{+}$ that is maintained close to $y^{+}=1$.

Considering the airfoil surface as a boundary affected by viscosity within the fluid domain, it is assumed that the fluid is incompressible, using the air as the gas model. The dimensionless wall distance $y^{+}$ is maintained close to $y^{+}=1$, as wall functions are not utilized to resolve the boundary region (see Figure 7).

In the simulations using the coarse, medium, and fine grids, the number of cells remained nearly unchanged, meaning that no actual grid refinement took place. As a result, there was no significant variation in grid resolution between these cases, and the aerodynamic coefficients ($C_{d}$ and $C_{l}$) remained constant across all grid levels. This suggests that the simulations had already reached convergence from the start, but it does not represent a traditional grid refinement study, where the number of cells typically increases progressively from coarse to fine.

For the simulation setup, a pressure-based solver was utilized with an absolute velocity formulation, ensuring accurate resolution of incompressible flow. The simulations were conducted in a steady-state mode, with a 2D planar space approximation to model the airfoil geometry and flow field. The inlet boundary condition was set with a uniform velocity of v=125 m/s, representing the freestream velocity. A pressure outlet boundary condition was applied to allow natural outflow.

Turbulent effects were modeled using the shear stress transport k-omega (SST k-omega) turbulence model, which provides accurate predictions for boundary layer behavior, especially in areas with adverse pressure gradients. A turbulent viscosity ratio was specified to capture the intensity and scale of turbulence in the flow. The solution method employed was a coupled solver, ensuring strong pressure–velocity coupling for faster convergence in steady simulations. Hybrid initialization was used to initialize the flow field, improving convergence speed and stability of the solution [55,56,57].

## 3. Results and Discussion

### 3.1. Analytical Method

**Figure 8:**
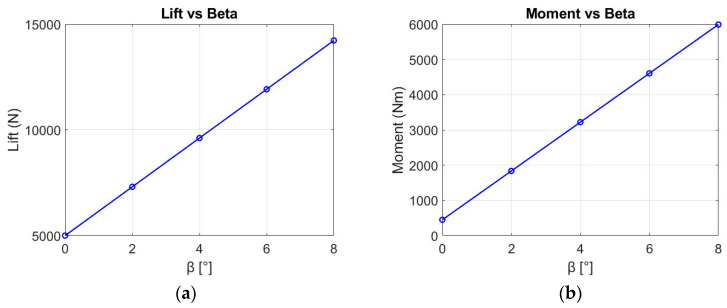
(**a**) Lift vs. β for NACA13112 airfoil using the analytical method; (**b**) moment vs. β for NACA13112 airfoil using the analytical method.

The additional lift generated by the control rotation is directed towards the hinge line of the trailing-edge flap, typically around two-thirds to three-quarters of the chord length. Consequently, any applied control rotation results not only in a lifting force inducing roll but also in a nose-down pitching moment, causing the blade’s AoA to decrease (see Figure 8).

The first plot represents the variation of lift as a function of β. As β increases, the lift increases. This behavior is expected because increasing β (either through flap deflection or morphing) effectively increases the camber of the airfoil. A higher camber leads to a stronger pressure differential across the airfoil, which increases the lift.

The second plot shows the variation in moment as a function of β. The moment is also linearly dependent on β in this range, showing that as the surface deflects, it generates a larger moment. This is typical because the deflection alters the pressure distribution along the airfoil, especially near the trailing edge, where it has a significant influence on pitching moment. The positive slope suggests an increasing nose-down moment as β increases, which is consistent with the behavior of most trailing edge control surfaces.

It is plausible to derive deductions regarding the influence of blade dimensions and material on its twist, and to establish principles aimed at enhancing its magnitude without causing deviations from the intended flight envelope. The diminishment of the gap among the center of aerodynamic pressure and the flexural axis, and/or the amplification of the GJ, leads to a rise in the magnitude of twist. If the flexural axis is positioned ahead of the aerodynamic center, the exerted aerodynamic moment becomes negative, resulting in a downward nose twist at the tip. When the flexural axis coincides with the axis of aerodynamic centers, aerodynamic loading does not induce any twist. Regrettably, these latter two design scenarios are typically impractical to implement in rotor blade designs, necessitating consistent consideration of twist for aeroelastic design, with sufficient GJ being vital.

### 3.2. Panel Method

**Figure 9:**
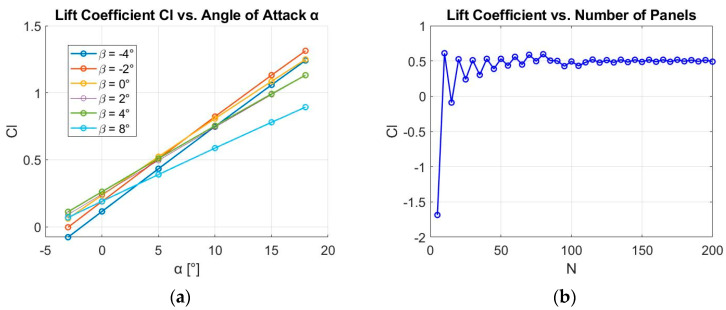
(**a**) $C_{l}$ vs. α for NACA13112 airfoil using the panel method; (**b**) $C_{l}$ vs. N for NACA13112 airfoil using the panel method.

Figure 9 illustrates the variation in $C_{l}$ with AoA, as well as the variation in $C_{l}$ with N, for the vortex panel method. Calculations were performed for β=[2°, 4°, 6°, 8°] at M=0.38 and α=10°. Comparing the two graphs, one obtained using the panel method and the other using CFD, several important aspects can be observed. Both graphs show a similar trend in terms of the increase in the $C_{l}$ with the AoA. As the AoA increases, $C_{l}$ also increases, which is expected in both CFD simulations and the panel method. It is notable that the inviscid solution furnished by the panel method gradually loses accuracy at higher AoA (α>15°) values due to viscous effects. In the CFD dataset, as the $C_{l}$ values reach their peak, denoted as $C_{l,max}$, the corresponding AoA designates the stall angle. Past this angle, while $C_{l}$ values from the panel method continue to rise, $C_{l}$ values from CFD begin to decline, ultimately leading to airfoil stall.

In general, the panel method may underestimate or fail to accurately capture boundary layer separation and other complex flow phenomena because it is inviscid and does not account for Reynolds number effects, viscosity, or turbulence—factors that are considered in CFD. However, it is still quite accurate for calculating $C_{l}$ in subsonic cases. In the presented results, the velocity was normalized to v=1 m/s to simplify the calculations. This works because $C_{l}$ is a dimensionless parameter and is primarily influenced by the airfoil configuration and AoA, not by the velocity itself.

The sensitivity of the results to N is shown in Figure 9b. It is evident that when N ranges from 10 to 100, $C_{l}$ is highly sensitive to N, indicating that the accuracy of the solution improves considerably as N increases. This is likely attributed to the fact that, at lower N values, the panel method lacks the resolution to adequately capture the curvature and flow distribution over the airfoil surface, leading to less accurate results.

However, as N increases beyond 100, the results become relatively insensitive to additional increases in N. This indicates that an adequate N value has been reached to accurately represent the airfoil’s geometry and flow characteristics. Beyond this point, further refinement of the panel count yields diminishing returns in terms of accuracy, as the numerical solution converges. Therefore, selecting an N value greater than 100 provides a balance between computational efficiency and accuracy, yielding reliable results without unnecessarily increasing computational cost.

**Figure 10:**
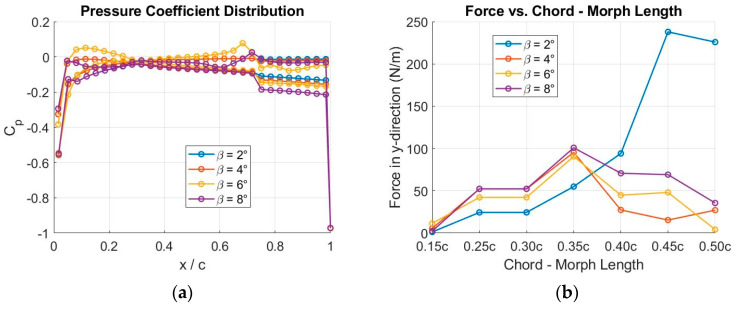
(**a**) The pressure distribution of the NACA13112 airfoil using the panel method; (**b**) change in force in the y-direction using the panel method.

Figure 10a presents $C_{p}$ distribution along the dimensionless cf of the NACA13112 airfoil for β=[2°, 4°, 6°, 8°]. The data, obtained using a linear vortex panel code, illustrate how the pressure distribution changes with increasing β. This analysis is crucial for determining the actuation force required to counterbalance aerodynamic loads at M=0.38 and α=10°.

The pressure distribution near the leading edge has a steep drop in $C_{p}$, which is characteristic of the leading edge suction that contributes to lift. The pressure stabilizes along the mid-chord and then rises sharply again near the trailing edge. As β increases from β=2° to β=8°, the overall pressure distribution shows a trend of increased pressure differences (lower $C_{p}$ values), particularly near the leading edge. This indicates that higher β values increase the lift produced by the airfoil. For β=8° the steepest gradient near the leading edge is observed, which suggests stronger suction and a more significant contribution to lift compared to the lower angles.

For all β values, the pressure coefficient remains relatively stable across a large portion of the airfoil (between cf=0.2c and approximately cf=0.7c), indicating a generally uniform pressure distribution in this region. However, a notable sharp rise in $C_{p}$ occurs near cf=0.75c, and significant differences in pressure distribution are most pronounced near both the leading and trailing edges. When the panel method is applied to an aerodynamic airfoil that is morphed with different flap–chord lengths (${cf}_{flap}$), high force concentrations appear in the morphed zone, as the changes in local geometry lead to variations in aerodynamic pressure on the airfoil surface. These pressure variations result in different forces acting on the airfoil, with higher concentrations in areas where the geometry has been significantly altered.

Figure 10b illustrates the variation of the force in the y-direction ($F_{y}$) acting on the lower surface of the airfoil in the morphed surface area for different ${cf}_{flap}$ values. The force is also plotted for β=[2°, 4°, 6°, 8°], with each case represented by distinct markers and colors. For all β values, the force increases gradually up to ${cf}_{flap}=0.35c$. However, beyond this point, distinct behaviors emerge. For β=2°, the force rises sharply after ${cf}_{flap}=0.40c$, peaking at approximately $F_{y}=240$ N/m near ${cf}_{flap}=0.50c$, indicating a dramatic increase in aerodynamic loading. In contrast, for β=[4°, 6°, 8°], the force begins to decrease after reaching ${cf}_{flap}=0.35c$, indicating that the force does not continuously increase with ${cf}_{flap}$ at higher β values. This suggests that for larger β values, the aerodynamic forces stabilize or reduce, while at lower β values, the force continues to increase significantly as ${cf}_{flap}$ increases. Overall, this figure highlights the nonlinear relationship between ${cf}_{flap}$ and aerodynamic forces for different β values.

### 3.3. CFD Method

**Figure 11:**
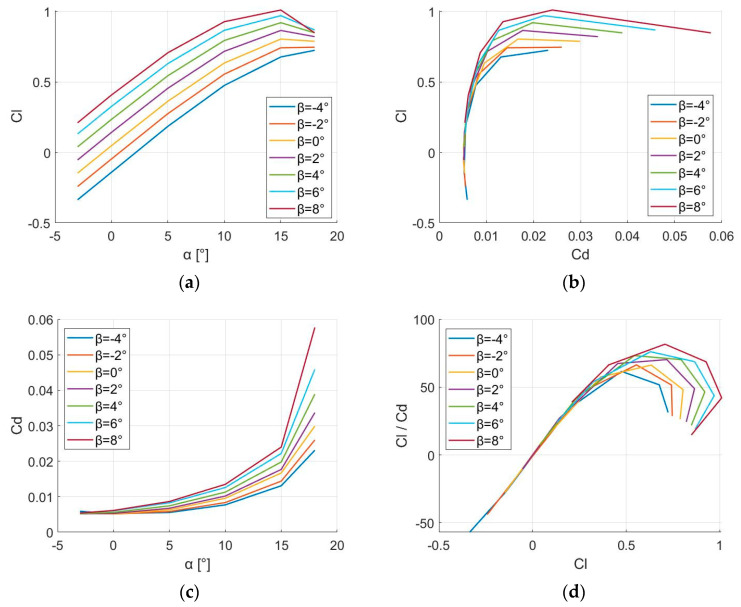
Results of aerodynamic analysis for different morphed configurations at M=0.38. (**a**) $C_{l}$ as a function of α; (**b**) $C_{l}$ as a function of $C_{d}$; (**c**) $C_{d}$ as a function of α; (**d**) $C_{l}$/$C_{d}$ as a function of $C_{l}$.

In Figure 11a–c, $C_{l}$ and $C_{d}$ coefficients for a range of morphed configurations are visually presented alongside those of the baseline configuration, showcasing their comparative behaviors. Both Figure 11a,b demonstrate a consistent trend of increased $C_{l,max}$ with higher β values. Additionally, the airfoil polars closely overlap for $C_{l,min}\leq C_{l}\leq C_{l,max}$, indicating that morphed airfoils generate more lift with a similar drag penalty as that associated with the baseline airfoil (see Figure 11).

To assess the aerodynamic effectiveness of the idea, the ratio of lift to drag is illustrated for various morphed configurations at M=0.38, as depicted in Figure 11d. It is clear that the aerodynamic effectiveness improves as β increases. This enhancement is particularly pronounced for $C_{l}>0.6$, especially at β=6° and β=8°.

TRIC employs movable components within the airfoil structure, specifically at the trailing edges. These components can be moved vertically, altering the camber of the airfoil. This movement is driven by mechanical actuators. By adjusting the camber in real-time, the airfoil can optimize its configuration for various flight phases, such as takeoff, cruising, or landing, which enhances lift and reduces drag. This leads to a higher lift-to-drag ratio, improving overall aerodynamic efficiency. Figure 11 illustrates how adjusting morphed camber can increase lift during hover flight.

**Figure 12:**
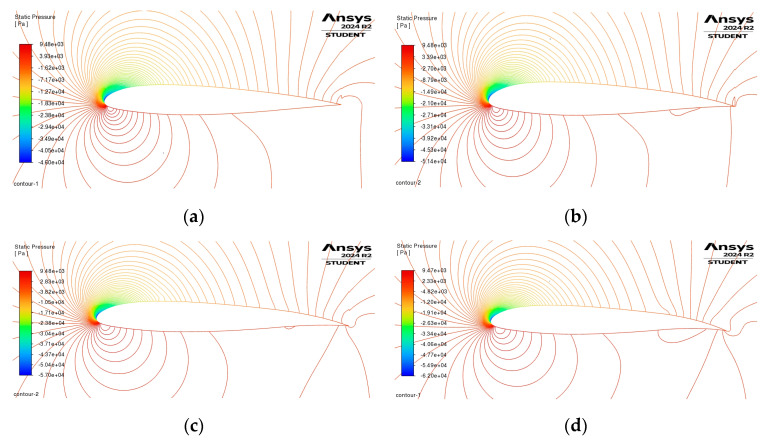
Here, we examine the contrast in pressure contours, normalized, between the camber morphing configurations and the standard airfoil, noted at M=0.38 and AoA α=10°: (**a**) β=2°; (**b**) β=4°; (**c**) β=6°; (**d**) β=8°.

A few flow visualizations using the SST k-omega model are depicted in Figure 12. With variations in β=[2°, 4°, 6°, 8°] at M=0.38, α=10°, and a flap–chord length of ${cf}_{flap}=0.25c$, it was observed that the baseline airfoil displayed lower pressure at the leading edge on the upper surface. This occurs because, when varying β within the range, there will be a moment that causes the leading edge to move downward, resulting in an increase in pressure on the upper surface in the leading edge area.

**Figure 13:**
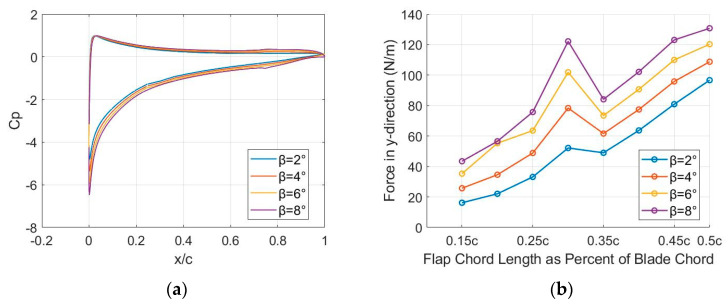
(**a**) Pressure distribution for NACA13112 airfoil when α=10° and β=[2°, 4°, 6°, 8°] using CFD; (**b**) force in the y-direction when α=10° and β=[2°, 4°, 6°, 8°] using CFD.

CFD can capture boundary layer separation at higher AoA or β values, which affects the pressure distribution near the trailing edge. This is especially important when the flow transitions from attached to separated, which can cause a steep drop in pressure near the trailing edge. The CFD result in Figure 13a likely shows more pronounced behavior near the leading and trailing edges due to separation effects that are not present in the panel method. CFD includes viscosity, which affects the pressure recovery along the chord, especially near the trailing edge. This is evident in Figure 13a, where the pressure distribution gradually changes, likely influenced by viscous drag and turbulent effects.

For a more thorough understanding regarding how the camber morphing configurations affect flow characteristics, the pressure coefficient, $C_{P}$, is depicted across the chord in Figure 13a. With the incorporation of camber alteration in the rear section, the adverse suction pressure increased downstream along the upper surface of the airfoil cf≈ 0.67c.

This phenomenon was particularly pronounced for the camber-modified airfoil with the most significant trailing-edge deflection. Such pressure augmentation exerted a significant influence on the boundary layer. Typically, the maximum $C_{l}$ of a plain flap is influenced by ${cf}_{flap}$. A flap–chord ratio ranging between ${cf}_{flap}=0.2c$ to ${cf}_{flap}=0.3c$ often results in the highest value of maximum lift. Nonetheless, it remains crucial to ascertain the impact of varying the flap–chord length around ${cf}_{flap}=0.25c$, as deviations from this ideal value are inevitable in certain scenarios.

Figure 13b reveals significant insights into the force distribution on the lower morphed surface of the aerodynamic airfoil. The force demonstrates a non-uniform variation between ${cf}_{flap}=[0.15c;0.50c]$ for β=[2°, 4°, 6°, 8°]. A distinct increase in force is observed at ${cf}_{flap}=0.30c$, after which the force follows a more consistent increasing trend, though with some non-linear characteristics. It can be observed that, as β increases, the force values become higher. These results can be attributed to changes in local geometry, which alter the pressure distribution and create higher pressure gradients in the morphed regions.

The higher AoA (α=10°) in the morphed area, as depicted in Figure 13, leads to increased pressure and, consequently, higher forces. This has practical implications for the aerodynamic performance, suggesting that while morphing can enhance control and lift, it may also introduce areas of high force concentration that need to be managed to avoid flow separation and ensure stability. For future calculations, ${cf}_{flap}$ will be adjusted accordingly if, for instance, the available volume for housing the actuation system proves to be insufficient. Therefore, CFD simulations were conducted for a flap deflection of β=[2°, 4°, 6°, 8°] to determine the actuation force required to counterbalance aerodynamic loads at M=0.38 and α=10°. These findings highlight the critical role of geometric modifications in aerodynamic behavior and provide a foundation for further optimization of morphed aerodynamic airfoils to achieve improved performance and stability.

To finalize the rotorcraft section design, several factors must be taken into account, such as operational frequency, dimensions, applied force, voltage requirements, weight, initial investment, control systems, and upkeep costs. The number of actuators needed and their corresponding weight for a given flap length and operational scenario is influenced by the force requirement. However, this study primarily concentrates on developing a morphing system with a focus on the principal criterion: actuation force. While other factors are crucial and should be assessed prior to the finalization of the section design, this study narrows its focus specifically to actuation force.

## 4. Conclusions

This article was dedicated to exploring the characterization of the morphing flap, based on the TRIC concept, of the NACA13112 airfoil. It introduced a design methodology where aerodynamic loads were integrated into CFD simulations to achieve the desired morphed airfoils. Additionally, factors influencing the configuration and actuation forces, such as ${cf}_{flap}$, were scrutinized to showcase the feasibility of making appropriate selections by considering actuation forces.

This computational exploration makes a substantial contribution to improving comprehension of the aerodynamics associated with camber morphing airfoils in 2D through CFD simulations. The camber modification was applied seamlessly, starting from ${cf}_{flap}=0.5c$ up to the trailing edge of the standard NACA13112 airfoil, with flap deflections ranging from β=[2°, 4°, 6°, 8°]. The analysis was conducted at M=0.38 for AoA values spanning α=[−3°, 18°].

CFD provides a more complete and realistic representation of the flow field since it incorporates both inviscid and viscous effects, as well as turbulence modeling. This makes it more suitable for cases where boundary layer separation, flow reattachment, or wake dynamics are important.

CFD captures complex flow features, like flow separation, pressure recovery, and the impact of turbulent flow on the pressure distribution. These are crucial for accurately predicting aerodynamic forces, especially at higher AoA values or when dealing with complex geometries.

The panel method is much faster and requires significantly less computational resources than CFD. This makes it useful for preliminary design studies or for cases where a quick estimation of aerodynamic properties is sufficient.

For low AoA values or simple, attached flow conditions, the panel method can give a reasonable estimate of the pressure distribution and overall lift without the need for complex and time-consuming simulation. It offers a balance between simplicity and accuracy, particularly in the early stages of design or for subsonic, attached flows.

Implementing continuous cambering along the trailing edge ${cf}_{flap}=0.25c$ of the baseline airfoil had a noticeable impact on the airfoil polars. This adjustment resulted in higher $C_{l}$ values for the same drag compared to the baseline airfoil. Elevating the camber resulted in a gradual rise in $C_{d}$.

When analyzing the pressure distribution, it became apparent that morphing the camber significantly affected the aerodynamic loads. Near the starting point of the segment with adjustable camber (i.e., ${cf}_{flap}=0.25c$), the positive pressure on the lower surface increased, while the adverse suction pressure on the upper side of the airfoil intensified as well. Pressure variations in close proximity to the trailing edge were notably accentuated with higher morphing deflections, leading to additional pitching moments.

The aerodynamic characteristics of the 2D airfoil will be used to compute the lift distribution along the span of the helicopter blade using the lifting-line theory, which models how lift is generated and distributed across the rotating blade. Induced by active camber actuation, variable elastic blade twist ramifications, along with the aerodynamic and structural loads, will be thoroughly analyzed to understand the influence of camber morphing airfoils on overall rotor performance. This comprehensive approach aims to foster an enhanced comprehension of these aspects.

Future research endeavors can build upon this study, utilizing it as a robust foundation. There is potential to develop a rapid and straightforward coupled aero-structural optimization model that integrates load-bearing components. In subsequent investigations, this algorithm can be readily expanded to optimizing entire rotors and other three-dimensional aerodynamic models, showcasing its adaptability and potential for further development. Additionally, the airfoil initially generated by this model can serve as a starting point, allowing for further refinement through the application of fluid–structure interaction and topological optimization techniques.

## Figures and Tables

**Table 1:** Aerodynamic characteristics of the rotor of the IAR 330 PUMA military helicopter [28].

Property	Unit	Value
Control method	-	Active
Airfoil type	-	NACA 13112 ^1^
Rotor radius	m	7.5
Blade chord	mm	600
Blade twist	-	8°37′
Rotation speed	rad/s	27.8
Advance ratio	-	0.42
Frequency	RPM	265
Pressure at sea level	Pa	101,325

^1^ The analyzed blade section.

**Table 2:** Resolution of the grid for the convergence study.

Airfoil	Resolution (Number of Cells)	
	Use Adaptive Sizing (No)	Use Adaptive Sizing (Yes)
Baseline	262,000	360,800
β=8°	319,200	360,000

## Data Availability

The original contributions presented in the study are included in the article, further inquiries can be directed to the corresponding authors.
